# Changing the COVID-19 Narrative in Africa: Using an Implementation Research Lens to Understand Successes and Plan for Challenges Ahead

**DOI:** 10.5334/aogh.3001

**Published:** 2020-08-19

**Authors:** Agnes Binagwaho, Miriam F. Frisch, Jovial Thomas Ntawukuriryayo, Lisa R. Hirschhorn

**Affiliations:** 1 University of Global Health Equity, Kigali, RW; 2 Feinberg School of Medicine, Northwestern University, Chicago, IL, US

## Abstract

Despite predictions that the number of deaths in Africa due to COVID-19 will reach 10 million, overall, the continent has reported relatively few cases compared to the rest of the world. Many African countries have been successful in containing initial outbreaks by rapidly using evidence-based interventions through implementation strategies adapted from other countries’ COVID-19 response as well as from prior epidemics. However, it is unclear whether these interventions will lead to long-term and complete success in stopping COVID-19 spread. Implementation research is a tool that can be used by countries to learn how to identify and understand contextual factors impacting COVID-19 prevention and control and select evidence-based interventions and strategies known to reduce spread of the virus. We identify seven key contextual factors that are facilitators or barriers to implementation of these interventions, and several strategies that can be leveraged if the factor is present or ones to strengthen if weak to improve implementation. These factors are: a culture of accountability, national coordination, financial stability of the population, culture of innovation, culture and capacity for research, health systems strength, and cross-border economies. Implementation science methods can serve to develop knowledge at a country and regional level on how to identify, utilize, and address these and other contextual factors, and inform relevant evidence-based interventions and implementation strategies. This approach can support African countries’ ability to address key challenges as they arise, both in fighting COVID-19 and future health systems challenges.

There has been concern internationally about the impact of COVID-19 on African countries. With the majority of the countries on the continent rated “least prepared” in their global health security prior to the pandemic, Africa’s health systems were expected to be quickly overwhelmed by COVID-19. Estimates put the number of projected deaths due to SARS-CoV-2 at approximately 10 million [1,2].

While countries across Africa are at different stages of their responses, overall, they have reported relatively few cases compared to the rest of the world [3]. As of June 6, there have been only 1.24 confirmed cases per 10,000 inhabitants across Africa on average [3]. Rates are highest in South Africa (7.75 cases per 10,000 inhabitants) and lowest in Lesotho (.02 cases per 10,000 inhabitants) (Table 1) [3]. Death rates are low as well, with only 0.03 deaths per 10,000 inhabitants across the continent [3]. On the same date, the US (ranked “most prepared” in global health security and reporting its first cases only a few weeks before Africa) had 57.67 cases and 3.35 deaths per 10,000 inhabitants; the UK, also ranked “most prepared,” had 43.06 cases and 6.12 deaths per 10,000 inhabitants [1,4].

**Table 1:** COVID-19 data across various countries and WHO regions [3,4].

Country/WHO Region	Number of Inhabitants	Date of the first COVID case	Number of days between the 1st case and June 6	Cases by June 6	Cases/10000 inhabitants by June 6	Deaths by June 6	Deaths/10000 inhabitants by June 6

UK	65,789,000	29-Jan-2020	129	283,315	43.06	40,261	6.12
Italy	59,430,000	31-Jan-2020	127	234,531	39.46	33,774	5.68
Spain	46,348,000	31-Jan-2020	127	240,978	51.99	27,134	5.85
US	322,180,000	19-Jan-2020	139	1,857,872	57.67	107,911	3.35
Canada	36,290,000	25-Jan-2020	133	94,070	25.92	7,652	2.11
South Africa	56,015,000	5-March-2020	93	43,434	7.75	908	0.16
Lesotho	2,204,000	13-May-2020	24	4	0.02	0	0.00
Rwanda	11,918,000	14-March-2020	84	420	0.35	2	0.002
Uganda	41,488,000	22-March-2020	76	686	0.17	0	0.00
Africa	1,019,922,000	14-Feb-2020	113	126,561	1.24	3,062	0.03
Americas	992,155,000	19-Jan-2020	139	3,155,370	31.80	176,167	1.78
Western Pacific	1,889,901,000	31-Dec-2019	158	189,030	1.00	7,092	0.04
South-East Asia	1,947,632,000	13-Jan-2020	145	336,577	1.73	9,316	0.05

This unexpected outcome in Africa is speculated to be due to a range of possibilities including limited testing capacity, poor reporting, younger populations, and differences in climate and humidity [5]. Some of these factors, especially limited testing capacity, are concerns across the continent [6]. These explanations do not tell the full story of the diverse and often successful responses across Africa to prevent the spread of COVID-19.

Many African countries have been successful in containing the initial outbreaks because they are rapidly and effectively implementing evidenced-based interventions including promoting and facilitating: handwashing, social distancing, testing, contact tracing, and lockdowns [7]. They have learned these interventions from Asian and European countries earlier on in the pandemic and from prior epidemics including SARS and Ebola. The difference from the previous epidemics and this pandemic is that Africa is adapting implementation strategies to reflect their own contextual factors or developing new implementation strategies appropriate to the country and region.

Rwanda and Uganda are two countries that took early and strong approaches before reporting their first cases of COVID-19. As a result, both countries have successfully prevented and mitigated the spread of the virus [3,8]. Rwanda began implementing evidence-based interventions including airport screenings in January 2020 [9]. These practices continued after Rwanda confirmed its first cases in mid-March, with systematic contact tracing according to World Health Organization (WHO) standards, and national lockdowns. Similarly, Uganda initiated preventive measures such as social distancing and travel restrictions four days before registering their first case, while implementing additional interventions including lockdowns once cases were confirmed [10]. The responses in these countries show that effective outbreak prevention is not due to the strength of the health system, but more the ability to identify and address the right contextual factors, with strong leadership, when rapidly and effectively implementing evidence-based interventions [11,12].

While African countries vary in their ability to contain the pandemic, there is a need to assess whether the high prevalence of asymptomatic and mild cases found in other parts of the world is also true in Africa [13]. Current data suggests that while there is now community spread and growing challenges in a number of countries in Africa, other countries have still continued to have success in preventing the widespread infection predicted [1,2].

## Implementation Research as a Tool to Understand and Maintain Success in COVID-19 Prevention and Control

It is unclear whether these prevention interventions, when effectively implemented, will lead to long-term and complete success in stopping COVID-19 spread. Interventions, including border closings and social distancing, can only go on so long. Reopening requires strategic and evidence-based actions. Evidence shows that even some countries with early successes are now struggling when community spread takes off, such as Ghana, which, as of June 6th, is experiencing a spike in infection after reporting lower case numbers since its first cases in mid-March [3].

Implementation research is a tool that can facilitate continued success for countries performing well. It provides methods to evaluate the use of strategies to integrate interventions into real-world settings to improve health outcomes, to understand the contextual factors that need to be addressed, and to inform adaptation of these strategies. This tool can also accelerate the production of knowledge needed to contain outbreaks in countries with less successful responses, while suggesting informed approaches to maintain pandemic control [11]. Using the methods in implementation research, countries can understand their successes and challenges by identifying not only what strategies to use, but also identifying how contextual factors can serve as either facilitators or barriers to implementation [14]. Countries can use that knowledge in their decisions to adapt strategies to implement evidence-based interventions, meet local needs, and inform effective and equitable infection prevention and control measures.

Following is a number of contextual factors that are emerging as important facilitators and barriers in response efforts. There are also outlined implementation strategies that can be chosen to address or mitigate factors if weak, and leverage when strong, for effective implementation of evidence-based interventions for pandemic response (and other recurrent outbreaks such as Ebola or cholera) as well as in maintaining primary care.

1. **Culture of Accountability:** The presence of an existing chain of accountability for any health threat is a major contextual factor to be regularly assessed for COVID-19. This culture will make implementation strategies such as developing national policies more effective and easier to adapt with new scientific findings and changes in the pandemic status. A culture of accountability can also facilitate the integration of these interventions into existing systems for sustainability [12].
2. **National coordination:** A culture of partner and sectoral coordination is a preexisting facilitating factor needed to develop a national COVID-19 response. Government leadership can build up a strong national coordination through using a multisectoral approach as a strategy to change this factor from a barrier to a facilitator. Ministries of health can strengthen communication with other ministries including labor, education, internal security, and immigration, to ensure effective and coordinated outbreak preparedness and response, and economic recovery. Strategies of donor coordination and stakeholder engagement can help facilitate a comprehensive implementation of evidence-based interventions, including to the most vulnerable populations, and to map and understand where resources are available (i.e. laboratory capacity to conduct tests or provide oxygen to patients) and where additional resources are needed. Integrating these strategies to build coordination can be further facilitated by a culture of accountability, which is important for successful and timely implementation.
3. **Financial stability of the population:** Populations living in poverty or employed through the informal economy need special consideration when additional lockdowns or plans for phased reopenings are made because of their limited financial safety nets and higher risk of COVID-19 due to their need to work outside the home. Governments need to acknowledge and address this contextual barrier by adapting implementation strategies such as a focus on equity and community engagement and providing support to reduce socioeconomic hardships related to COVID-19 response. Ignoring this factor, however, can result in an inability for populations to adhere to lockdowns or strict social distancing interventions.
4. **Culture of Innovation:** The growing culture of innovation across the African continent is a facilitating contextual factor that can be leveraged as part of response efforts through implementation strategies such as support for rapid development, early adoption, and scale-up of innovations. Innovations that have recently been developed, or are in development throughout the COVID-19 pandemic, include low-cost rapid testing kits, web-based alerting systems to counter misinformation, and locally manufactured ventilators, all to meet the needs of low-resource settings [15]. This culture can be leveraged through the use of community health workers by providing them with appropriate protection and training for education and engagement.
5. **Culture and Capacity of Research:** The lack of investment in building a culture of research remains a barrier in many countries. Strong internal research capacity is important for generating context-specific understanding and adaptations of both existing and new evidence-based interventions, such as those for phased re-opening of society. Identifying areas where local research is needed, drawing on the existing research capacity, and building new research capacities are strategies which can accelerate the most effective response for pandemic recovery, and prepare for future health system shocks. Generating and using data for decision making and focusing on equity also helps guide policy decisions to address the needs of the most vulnerable while improving population compliance with prevention and control guidelines.
6. **Strength of the Health Sector:** Health systems in sub-Saharan Africa do not possess the resources or resilience of those in the Western world, that still struggle to control COVID-19 outbreaks [1,3]. The health of populations, especially those with co-morbidities such as malnutrition, tuberculosis, or HIV, are expected to suffer as a result of the pandemic. For example, with interventions such as lockdowns, some patients are unable to access transportation to get medication or needed treatment; on top of limited transportation, systems may not be in place for televisits or home delivery of medications. Further, weak health systems limit the capacity to care for critically ill patients with COVID-19 while maintaining primary care services during an outbreak. Unless implementation strategies are chosen and adapted to address weak health systems, there could be dramatic health repercussions, including increasing under-five and maternal mortality rates, decreasing vaccination rates, and limited access to health care [16]. Strategies to mitigate the weaknesses of the health sector include task shifting from clinicians to community health workers, community based education and engagement (especially where community health systems are strong) and leveraging existing systems for implementation.
7. **Cross-border Economies:** The need to transport goods and workers regionally is a major contextual factor for implementation of evidence-based interventions. Preventive measures such as border closings between countries with different levels of COVID-19 create a major barrier for a successful implementation measures. For example, despite strict lockdowns and limited spread, Rwanda reported a jump in the number of cases since early April, due to truck drivers infected with COVID-19 crossing the border from Tanzania, a country without social distancing or lockdown policies [9,17]. Strategies of international and multisectoral collaboration are key to addressing this issue. Institutions such as the African Union and East African Community need to influence, develop, and sustain regional and national responses while encouraging dialogue and alignment of cross-national policies and regulations. While the East African Community has brought together heads of states and cabinet members with varying level of success, more needs to be done to improve collaboration and supra-national coordination [18]. Collaboration as a strategy will be crucial to facilitate successful border openings and allow countries to quickly adapt based on changes to COVID-19 spread.

As the world begins to see subsequent waves of COVID-19, and before an affordable treatment or vaccine is available, an implementation research approach to understanding, responding to, and addressing contextual factors will be needed more than ever. African countries need to learn at the national and regional levels how to acknowledge contextual factors. As key challenges arise, they need to also develop the capacity to carefully identify and adapt relevant implementation strategies reflecting these factors, including a focus on equity, using and leveraging existing systems, international and multisectoral collaboration, and data gathering and interpretation to inform decision-making. This ongoing learning will be critical in determining the approach and timing of a phased re-opening or in responding to increases in cases and changes in local pandemic patterns of spread.

In conclusion, implementation research offers policy makers, implementers, and public health professionals the tools to identify contextual factors, choose relevant strategies, measure successes and failures, and continue to adapt and learn. Implementation research can also help generate broader knowledge useful to other countries facing similar challenges while sustaining stronger work from ongoing health systems. This knowledge is important in reducing needless mortality from COVID-19 and future pandemics; at the same time, eliminating broader barriers to quality, equitable healthcare for all.
